# Vaccination distribution by community pharmacists under the COVID-19 vaccine appointment system in Taiwan

**DOI:** 10.1186/s12962-021-00331-2

**Published:** 2021-11-22

**Authors:** Ya Wen Lin, Che Huei Lin, Ming Hung Lin

**Affiliations:** 1 Department of College Public Health, China Medical University, Taichung, Taiwan; 2 School of Nursing, China Medical University, Taichung, 40402 Taiwan; 3 Department of Nursing, National Taichung University of Science and Technology, Taichung, Taiwan; 4 College of Pharmacy and Science, Chia Nan University of Pharmacy & Science, Tainan, Taiwan

**Keywords:** Pharmacists, COVID-19 pandemic, Community pharmacy, Vaccine

## Abstract

Pharmacists play a critical role in implementing and promoting public health policies, particularly during pandemics, thanks to their exceptional skills, knowledge, expertise, and accessibility to the public. This study aimed to increase the roll-out of COVID-19 vaccines in a coordinated manner to ensure equal accessibility to all Taiwanese residents. A total of 3301 health insurance special pharmacies, equivalent to 80% of all community pharmacies in Taiwan, are assisting the public in scheduling vaccines. Once pharmacists had ensured adequate vaccine availability, vaccinations were scheduled depending on the time of registration on the platform for vaccination appointments. The roll-out of this program saw a significant number of people register and receive the vaccine throughout the country, and the number of individuals who received both a first and second dose increased significantly. Community pharmacy-based distribution of the vaccine to the public signifies the novel and innovative involvement of pharmacists in government initiatives to promote public health. Our study shows that community pharmacies can potentially enhance the efficiency and equitable distribution of health supplies and resources, particularly during pandemics.

## Introduction

Achieving primary healthcare and enhancing people-centered healthcare services necessitates the involvement of professionals across the entire healthcare spectrum as well as the community. The key to ensuring an informed public health management strategy is to involve and empower entire communities so that individuals take responsibility for their own health and that of other community members. Gilmore et al. emphasized that community engagement can effectively support sustainability as well as the buy-in of healthcare interventions, availability of improved healthcare services, health advocacy, and improved satisfaction of care services, with a concomitant promotion of responsiveness and strength within healthcare systems. Community engagement is broadly defined as the individual and group participation of community members within the confines of a community’s social boundary to ascertain effective decision making, designing, planning, governance, and provision of services [1]. Terms such as social mobilization, communication, community action, community participation, and empowerment stress the agency of groups of individual members by considering them to be active rather than passive members.

With healthcare centers almost overwhelmed by extraordinary demand in terms of patient admissions and treating a large number of COVID-19 cases, pharmacists were required to adopt and adapt to changes in their professional role amidst scarcity of resources [1]. Previous pharmacy-based community programs rolled out across the country have revealed pharmacists’ contributions in facilitating the distribution of limited public health management resources to community members.

Pharmacists in Taiwan have set up many pharmacy-based programs such as the “COVID-19 vaccine registration and appointment reservation system,” which has shown the indisputable importance of the active involvement of pharmacists affiliated to the country’s National Health Insurance in implementing public health promotion strategies. Pharmacists have been commended for contributing toward achieving optimized outcomes and for providing an avenue to connect with and improve society in cost-effective and reliable ways. Ensuring a healthy population may not be possible if the healthcare system does not commit to implementing and promoting informed public health strategies via the participation of all individuals, groups, and public health professionals [2]. Therefore, investing in innovative public health management strategies that ensure the sustainability and competitive performance of Taiwan’s healthcare system is essential.

Pharmacists possess valuable knowledge, expertise, and skills required to ensure reasonable implementation of the health promotion strategies rolled out by the government. Notwithstanding their point of entry into the healthcare system, pharmacists are an invaluable element of the conventional health service utilization trend and the adoption behavior of community members for health services [3]. Pharmacists’ strategic involvement and contribution to embracing public health promotion strategies partly derive from their proximity advantage. In Taiwan, community pharmacists can assist with the implementation of many of the government’s public health initiatives such as the “Mask Real-Name System,” which is meant to ensure uniform distribution of face masks to individuals and families throughout the country.

Elsewhere, pharmacists have also contributed significantly to caring for COVID-19 patients by facilitating effective clinical trials, promoting information sourcing and sharing, and providing appraisals for enhancing evidence-based practice, providing routine health services, and imparting health education to the public [4].

Since February 2020, the COVID-19 epidemic has spread unremittingly throughout the world. Several approaches have been adopted by many countries, including Taiwan, to control the spread and severity of the pandemic, including the development and testing of vaccines produced by companies such as Moderna, AstraZeneca, and BNT. While developing an effective vaccine constitutes a critical step toward minimizing the severity of the threat posed by COVID-19 to the public, ensuring a reasonable vaccination rate is also essential for controlling its spread. In order to achieve herd immunity, an estimated 67% of the population of Taiwan will need to be vaccinated [5], but this has not yet been achieved. Early this year, Chen Shih-Chung, the Taiwanese Health Minister, warned that the government was likely to face challenges in ensuring equal distribution of vaccines to the entire population, meaning there would be an “imbalance” between individuals who have been protected and those who are still at risk [6].

Vaccine shortages have often been cited as the main obstacle to achieving a reasonable rate of vaccination in Taiwan. Additionally, Taiwan’s conventional healthcare facilities have also failed to ensure that the country’s approximately 24 million inhabitants have been vaccinated within a reasonable timeframe. Consequently, as of May 22, 2021, only 299,844 individuals had received one dose of the vaccine, equivalent to just 1.3% of the population. Three months later, about 165,287,232 people in Taiwan had received one dose of the vaccine, translating to only 7% of the country’s total population, with only 29,704 having been fully vaccinated [7].

These figures show that the Taiwanese government and related agencies such as the Central Epidemic Command Center (CECC) and the National Health Insurance (NHI) need to urgently speed up the vaccination process in order to ensure the entire population will eventually be vaccinated. To increase vaccination uptake, the CECC began trialing a COVID-19 vaccination appointment system in Penghu, Matsu, and the Kinmen Islands before rolling out the system throughout the country. The system was developed by Audrey Tang, a designated Minister without Portfolio. After discussion with local governments, the system was used for scheduling vaccination appointments for anyone under 65 years old. Thus, from July 6 (10 a.m.) to July 7, 2021 (5 p.m.), the residents from the three islands belonging to categories 9 and 10 on the vaccine priority list could log into the system website (1922.gov.tw) using their residence permit numbers, national ID numbers, or NHI card numbers, and select their preferred vaccine brand and vaccination center [8].

On July 15, 2021, the CECC announced its plan to roll out the COVID-19 vaccine to eligible individuals who had already submitted their registration and booked for the second round of vaccination on the COVID-19 vaccination registration and appointment reservation systems [9]. As of July 6, 2021, at 2 p.m., more than 2,290 individuals belonging to categories 9 and 10 living in the three islands registered on the website’s system submitted their preferences, and 834,780 individuals residing in other parts of Taiwan did the same [10]. On the same day, by 10 a.m., about 2,628,789 people had received a dose of either the AstraZeneca or Moderna vaccine. Of the total administered doses, 2,581,047 (98.2%) were first doses and 47,742 were second doses, equivalent to 1.8% of the total number of doses [7].

**Fig. 1:**
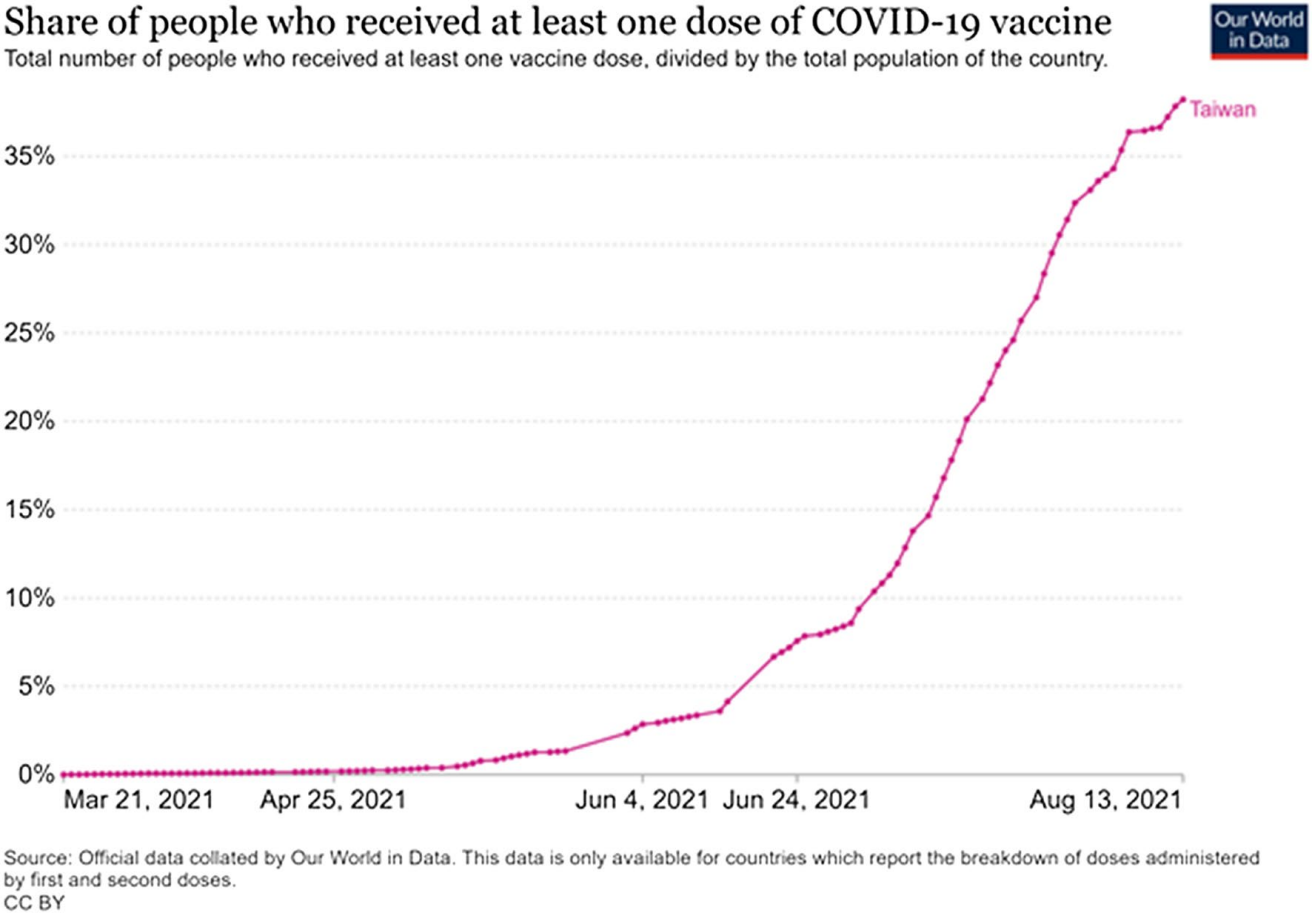
Share of people who received at least one dose of COVID-19 vaccine in Taiwan. Data source: https://github.com/owid/covid-19-data/tree/master/public/data/vaccinations/locations.csv

**Fig. 2:**
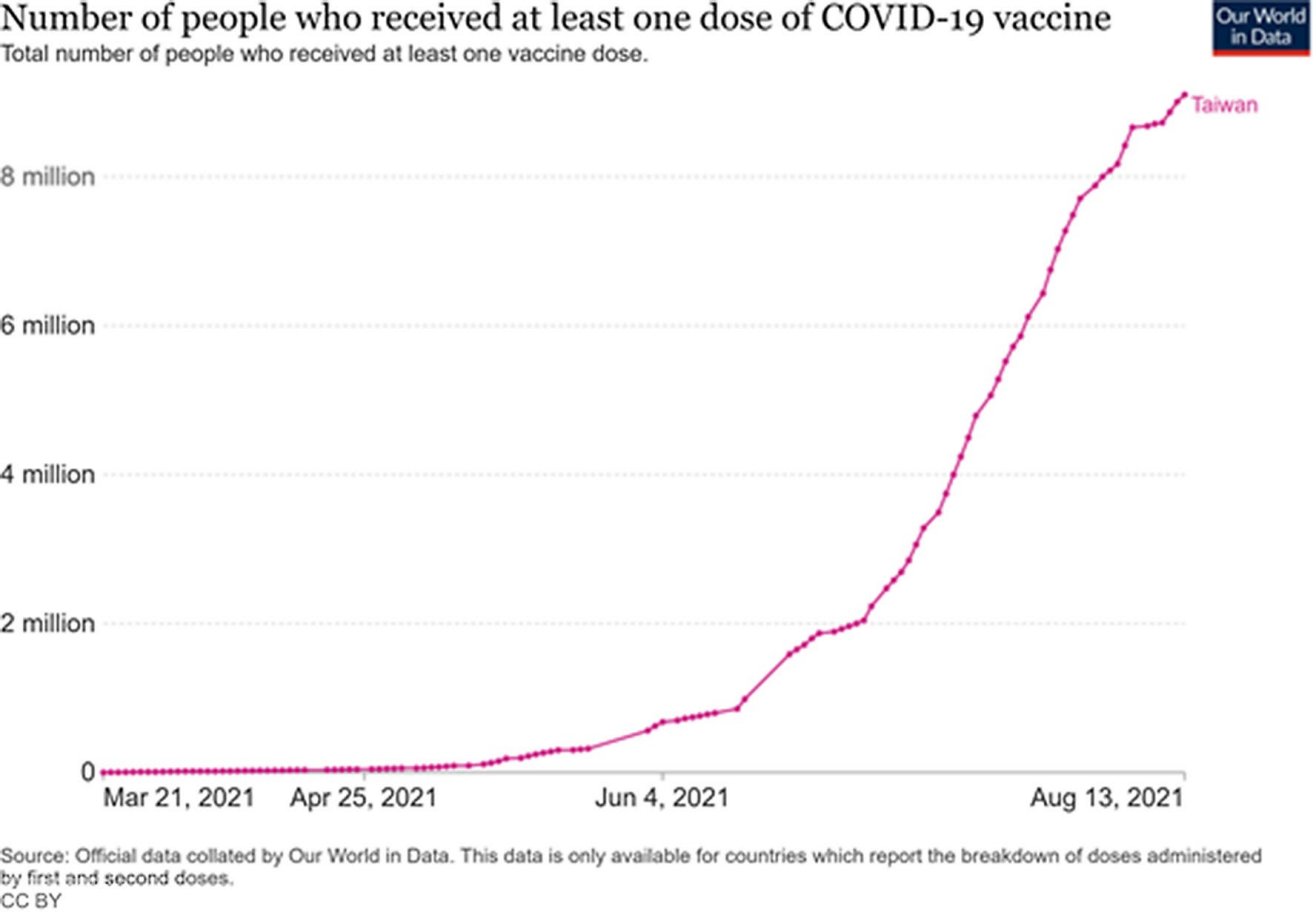
Number of people who received at least one dose of COVID-19 vaccine in Taiwan. Data source: https://github.com/owid/covid-19-data/tree/master/public/data/vaccinations/locations.csv

Although the special health insurance pharmacists were not directly involved in supplying the vaccine, their involvement significantly improved the effectiveness of the national public health initiative implemented to improve vaccine uptake. As of Aug 15, about 9,800,000 doses of the vaccine had been administered throughout the country (Figs. 1, 2), equivalent to 39% of Taiwan’s total population; of these, 649,000 people had been fully vaccinated, representing almost 2.76% of the country’s total population [7]. Given their effectiveness and exceptional performance record in helping the Taiwanese government implement public health promotion initiatives, special health insurance pharmacies were enlisted to facilitate booking vaccine appointments and administer vaccines to individuals in various categories throughout the country (Appendix 2 and 3). The primary objective was to ensure efficiency, robustness, and fairness when distributing COVID-19 vaccines among the priority groups.

### Program description

The ease in rolling out the “Registration and Appointment System for COVID-19 Vaccine Intentions” nationwide was attributable to the country’s high coverage rate for NHI (99%) and having a wider-ranging health insurance database than any other country in the world. Cumulatively, 3301 special health insurance pharmacies in Taiwan have joined the national vaccine team to assist with booking vaccination appointments, which translates to 80% of the national tally of special health insurance pharmacies [9]. Priority individuals needed to follow four steps: registration, waiting for contact via a text message through the 1922 hotline, booking a slot for vaccination, and receiving a vaccine shot at their preferred community pharmacy.

After logging in to the system using their residence permit numbers, national ID numbers, or NHI card numbers, individuals would then identify their preferred vaccine brand and vaccine center. Individuals belonging to priority category 9, and those born before 1992 when the Taiwan was established, could register from July 13th in the morning until July 15th at 5 pm [11]. Eligible applicants were notified by text message sent through the 1922 hotline that they were eligible to receive a vaccine shot the following week. After this, the applicants had to log on to the 1922.gov.tw website between Thursday and Sunday of that week to book an appointment at their preferred community pharmacy. The reservation process necessitated the use of NHI card or password methods, which required a card reader. However, if an eligible applicant was not interested or not in a position to use a card reader, they could visit a nearby pharmacy to book a time and location for their shot [12]. Several vaccine brands, including Moderna and AstraZeneca, would be available in community pharmacies for all eligible residents.

A rotational weekly based approach was adopted when offering the vaccines, meaning, for example, that if the AstraZeneca vaccine was being offered in week 1, the Moderna vaccine would be offered in week 2 or a subsequent week [10].

In order to avoid vaccine shortages, and to ensure that all eligible individuals received their preferred vaccine, pharmacists working in community pharmacies analyzed registration data every Wednesday at 5 pm. This entailed estimating the available vaccine doses and establishing the number of people in priority groups eligible to receive a vaccine the following week. Data analysis findings enabled both community pharmacies and local governments to note the preferences of each eligible recipient and to ensure sufficient availability [8]. If their preferred vaccine was available, eligible recipients were sent text messages prompting them to book an official appointment. The three options by which they could schedule an official vaccination appointment were booking through the website, using the NHI app, or visiting a community pharmacy [10]. According to Taiwan’s Pandemic Epidemic Command Center, more than 8.2 million Taiwanese citizens had finalized their vaccine registration as of July 28, 2021, at 1 pm. About half of the applicants preferred either the Moderna or AstraZeneca brand. For example, the AstraZeneca vaccine was requested by 39% of the applicants, while the mRNA vaccine (CNA (a) par 5) was requested by just 4.98% [13].

Individuals receiving their first dose were asked to show their NHI card or national ID, while those getting their second or third doses were simply asked to provide their COVID-19 immunization record [9]. Individuals who had been vaccinated would receive an official yellow card that records their vaccination information (Appendix 1).

Eligible recipients were expected to arrive at their preferred community pharmacy at their allotted time to avoid overcrowding. Although late arrivals would still be vaccinated, those who failed to show up on the appointed day were required to reschedule for the next week [8].

Successfully implementing the “Registration and Appointment System for COVID-19 Vaccine Intentions” necessitated close collaboration between community pharmacists, relevant government ministries, local governments, information technologists, finance personnel, and distributors of imported COVID-19 vaccines. According to the National Federation of Pharmacists Association of Taiwan, pharmacists have been working with the government and contributing significantly to the battle against COVID-19 for more than a year. Consequently, pharmacists were expected to continue to be involved in the vaccination programs [9]. In addition, the Yuan Executive requested the assistance of the National Federation in contacting special health insurance pharmacies previously involved in implementing the “Mask Real-Name System [16]” to be part of the national vaccine team to help the public book vaccination appointments.

Applicant eligibility was determined based on priority, with the public being grouped into various categories, of which priority was given to categories 9 and 10. Category 9 included people aged 18–64 who suffered from underlying health conditions that increased their risk of having a severe COVID-19 infection, while category 10 included individuals aged between 50 and 64 [10]. Furthermore, individuals suffering from terminal disorders and rare diseases could be easily identified since they had medical certification, whereas NHI medical records were used to identify individuals with serious illnesses [9].

Each eligible applicant could specify the hour they could receive their shot. Therefore, it was projected that vaccine administration would become more efficient by ensuring proper planning and coordination of the entire vaccination program.

The vaccine reservation program aimed to reduce delays, queuing, and the lack of equality when administering the vaccines. The vaccination appointment system would allow community pharmacists to easily evaluate the eligibility of applicants by reviewing their applications as well as the NHI database. Before eligible individuals were allowed to make an appointment, pharmacists analyzed the registration data to ensure that each person received their preferred vaccine. In actualizing this, pharmacists from selected community pharmacies in collaboration with local governments were required to consider the number of registrations and compare them with the number of available vaccination doses and brands available. This ensured that the available stock was enough to meet demand for the following week. With proper vaccination scheduling, pharmacists could plan ahead to ensure adequate staffing levels and resources to administer vaccine shots effectively and efficiently, thereby avoiding frustration due to delays or insufficient doses.

Community pharmacists helped expedite the vaccine administration process and continue to help the Taiwanese government toward achieving its goal of vaccinating the entire population. Although using the vaccination reservation system could have been challenging for elderly people with no one to assist them, the system facilitated equal vaccine administration and easy scheduling of vaccine doses to ensure that priority groups were fully vaccinated within their designated timeslots.

Initially, people who received the initial vaccination would often fail to return for their second and third doses. However, by using the system to notify and schedule applicants for follow-up vaccinations, administering the second and third doses became easier. This was made possible through the NHI Cloud system that helped pharmacists to identify and inform individuals requiring second and third doses of COVID-19 doses by reviewing their registration and vaccination data.

## Discussion

Because of Taiwan’s political status, it is difficult for the government to obtain vaccines. Consequently, the community pharmacy-based method for booking and administering COVID-19 vaccines to the highest priority groups has shown the importance of involving pharmacists in promoting efficiency, reliability, and equitability, and in coordinating the implementation of the government’s public health promotion initiatives. Through their involvement in electronic registration and online booking of vaccine appointments, community pharmacies have provided the NHI and the CECC with reliable mechanisms of ensuring equality and uniformity when administering the vaccine. Due to their proximity, community pharmacists have ensured that vaccines were readily available and accessible to those who would otherwise have to travel long distances to get inoculated or who were unaware of either what vaccines were available or where their nearest vaccination center was. The approach affords a swift mechanism of acquiring and sharing critical data that the government can use to make decisions and plan sourcing and distribution of the vaccine to ensure all eligible individuals are fully vaccinated within a reasonable timeframe. Specifically, this approach has ensured uniform availability of the vaccine by eliminating the possibility that certain individuals either may not receive all the required doses to become fully vaccinated or may not be vaccinated at all.

**Fig. 3:**
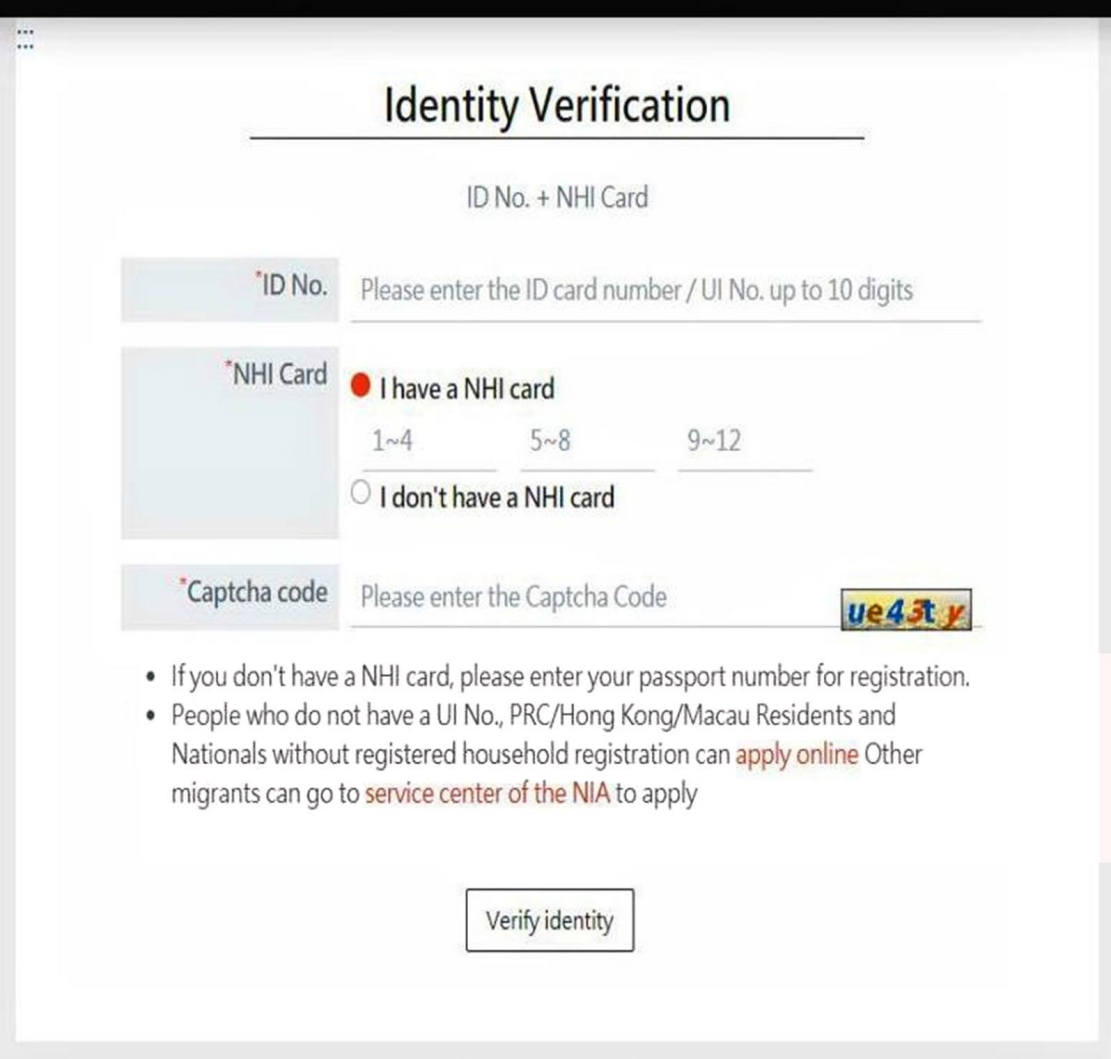
COVID-19 vaccination registration and appointment reservation platform. Data source: Taiwan Center for Disease Control, 2021

The Taiwanese online vaccination reservation platform website (Fig. 3) is publicly funded [13] and is easily accessible to all Taiwanese citizens and foreign nationals with NHI, although registration has been limited to priority groups. This method is easy and reliable because it is accessible online. When making an appointment, eligible individuals can either use the online system or the Health Insurance Auto toll app, available to both Android and iOS users, which has further increased accessibility and convenience.

Taiwan is a tech-savvy nation, with more than 97% of the population reportedly owning a smartphone [14]. Nevertheless, the system requires the use of a card reader, particularly for applicants using NHI cards, which will be inconvenient for people who do not have a card reader. Despite this shortcoming, the online COVID-19 vaccination booking system has revolutionized the vaccine administration process by introducing the aspect of online registration and appointment booking. This, in turn, could lead to less queuing at the vaccination centers. Although the vaccination reservation system is in Mandarin Chinese, the Ministry of Labor has also uploaded guidelines on the registration and reservation process in Vietnamese, English, Thai, and Indonesian [12]. By doing this, the Ministry of Labor helped resolve misinformation and miscommunication problems previously experienced by the CECC in its real-time public communication about the coronavirus public health crisis, which was conducted entirely in Mandarin Chinese and sign language (15).

Since March 2021, Taiwan has received several batches of COVID-19 vaccines from different sources and more are expected in the future. The vaccination reservation platform has led to many benefits, such as promoting vaccination appointment efficiency and boosting vaccine roll-outs to the public. The system has also ensured increased accessibility to the vaccine. However, administering vaccinations in community pharmacies has led to certain drawbacks, such as pharmacists becoming overworked as they have to balance their traditional prescription roles with vaccine administration. Although the Taiwanese government has provided much of the necessary support and services, community pharmacies may have to incur additional costs to ensure the necessary resources and personnel for administering vaccines.

Additionally, as the COVID-19 vaccination registration and appointment reservation system is linked to the NHI database, and perhaps to patients’ electronic health records throughout the country, community pharmacists are able to identify and write prescriptions for people with chronic health conditions.

In general, the involvement of pharmacists in the delivery of the COVID-19 vaccines has helped to reduce overcrowding in hospitals by greatly increasing the number of individuals receiving vaccines. Easing overcrowding and discouraging unnecessary travel, in turn, prevents the further spread of COVID-19. It turns out, therefore, that community pharmacists play a critical role in ensuring the sustainability and reliability of Taiwan’s national health system when handling a pandemic.

## Conclusion

To ensure effective management of the COVID-19 pandemic, the Taiwanese government is benefiting from a combination of community pharmacists and its comprehensive NHI information system to ensure thorough vaccine penetration into every community. The practice of empowering pharmacists to administer vaccines to priority groups as an approach for addressing the severity of the COVID-19 pandemic is an innovative public health management program. It can enhance the resourcefulness of pharmacists and their well-established presence in communities coupled with increased adoption of technology to ensure members of the public are reached in an efficient, reliable, and equitable manner. Although not without shortcomings, the publicly funded vaccine appointment platform further demonstrates how pharmacists can benefit public health initiatives by collaborating with the government and other organizations to ensure the increased roll-out of the COVID-19 vaccine with a well-coordinated distribution program that can reach the entire population. Furthermore, the program utilizes technology and allows pharmacists to gain lucrative returns by participating in initiatives to manage public health. The Central Epidemic Command Center (CECC) on Oct. 28, 2021 announced that the Taiwan first-dose vaccination rate had surpassed 70%, while its full vaccine rate had passed 30%, both ahead of the month’s end goal (Appendix 4), The CECC pointed out that Taiwan has achieved its goal of reaching 70% first-dose and 30% two-dose vaccination rates by the end of October ahead of schedule.

## Data Availability

Not applicable.

## Appendix 1

### Taiwan vaccination yellow card

Individuals who have received the vaccine would receive an official yellow card, as displayed above, that records their vaccination information.

Data source: https://www.cdc.gov.tw/File/Get/xgvRuwoMkPrZHZYTQ3sKXQ.

## Appendix 2

### Central Epidemic Command Center lists ten priority groups, with the first three to receive doses immediately (data source, Taiwan Centers for Disease Control)

Group 1: Healthcare workers.

Group 2: Central and local government epidemic prevention personnel.

Group 3: Frontline workers at high risk of coming into contact with COVID-19.

Group 4: People who need to travel abroad.

Group 5: Law enforcement officers and firefighters.

Group 6: Volunteers, long-term care workers, and care recipients at social welfare organizations.

Group 7: National security personnel.

Group 8: Adults aged 65 and above.

Group 9: Adults aged 19–64 with life-threatening conditions, rare diseases, or a history of serious illness.

Group 10: Adults aged 50–64.

## Appendix 3

### Central Epidemic Command Center lists ten priority groups

Data source: Central Epidemic Command Center https://data.cdc.gov.tw/en/dataset/covid19_tw__stats.

## Appendix 4

### CECC achieves ‘70%/30%’ goal ahead of 28, Oct 2021 end deadline

Data source: The Taiwan Central Epidemic Command Center (CECC), Taiwan News, Yuwen Lin.

## References

[1] Gilmore B, Ndejjo R, Tchetchia A, Tchetchia V, Mago E, Diallo AA (2020). Community engagement for COVID-19 prevention and control: a rapid evidence synthesis. BMJ Glob Health.

[2] Hayden JC, Parkin R (2020). The challenges of COVID-19 for community pharmacists and opportunities for the future. Irish J Psycholog Med.

[3] Anderson C. Pharmaceutical care, health promotion, and disease prevention. In: Alves da Costa F, Foppe van Mil JW, Alvarez-Risco A, editors. The pharmacist guide to implementing pharmaceutical care. 2019. p. 287–93. Cham: Springer, 287.

[4] Paudyal V, Fialová D, Henman MC, Hazen A, Okuyan B, Lutters M (2021). Pharmacists’ involvement in COVID-19 vaccination across Europe: a situational analysis of current practice and policy. Int J Clin Pharm.

[5] Randolph H, Barreiro L (2020). Herd immunity: understanding COVID-19. Immunity.

[6] Jennings R. Taiwanese health official warns against reliance on coronavirus vaccines [Internet]. 2021 Available from: https://www.voanews.com/covid-19-pandemic/taiwanese-health-official-warns-against-reliance-coronavirus-vaccines.

[7] Our World in Data. Statistics and research: Coronavirus (COVID-19) vaccinations [Internet]. 2021 Available from: https://ourworldindata.org/covid-vaccinations?country=~TWN.

[8] Lee IC. Vaccination booking system trial begins [Internet]. 2021 Available from: https://www.taipeitimes.com/News/front/archives/2021/07/07/2003760419.

[9] Taiwan Centers for Disease Control (Taiwan CDC). Starting July 16, vaccine to be administered to eligible recipients who scheduled appointments on COVID-19 government-funded vaccination appointment reservation system in the second round of registration; Third round of registration for vaccination appointments to be extended to 12 pm. on July 19 [Internet]. 2021 Available from: https://www.cdc.gov.tw/En/Bulletin/Detail/Gn-TIERW6HQL0awMxHuTkg?typeid=158.

[10] Chieh-Ling et al. Coronavirus/trial run of COVID-19 vaccine appointment system launched [Internet]. 2021 Available from: https://focustaiwan.tw/society/202107060014.

[11] Central News Agency (CNA) (b). Tang Feng plans to understand the operation process of the vaccine reservation platform online [Internet]. 2021 Available from: https://www.cna.com.tw/news/firstnews/202107065005.aspx.

[12] Everington K. English guide shows steps for Taiwan’s vaccine registration platform [Internet]. 2021 Available from: https://www.taiwannews.com.tw/en/news/4249001.

[13] China News Agency (CNA) (a). Taiwan’s local vaccines open to register their willingness to administer the public’s doubts and react indifferently [Internet]. 2021 Available from: https://www.tellerreport.com/news/2021-07-28-taiwan-s-local-vaccines-open-to-register-their-willingness-to-administer-the-public-s-doubts-and-react-indifferently.S1z-flCACu.html.

[14] Yan. Over 97% of people in Taiwan have smartphones: Report [Internet]. 2021 Available from: http://www.xinhuanet.com/english/2019-03/24/c_137920637.htm.

[15] Wang CJ, Ng CY, Brook RH (2020). Response to COVID-19 in Taiwan: big data analytics, new technology, and proactive testing. JAMA.

[16] Lin CH, Lin YW, Wang JY, Lin MH. The pharmaceutical practice of mask distribution by pharmacists in Taiwan's community pharmacies under the Mask Real-Name System, in response to the COVID-19 outbreak. Cost Eff Resour Alloc. 2020; 18(45). 10.1186/s12962-020-00239. 10.1186/s12962-020-00239-3 PMC7570415 33088224

